# Model organisms for functional validation in genetic renal disease

**DOI:** 10.1515/medgen-2022-2162

**Published:** 2022-11-29

**Authors:** Susanne Boettcher, Matias Simons

**Affiliations:** Sektion Nephrogenetik, Institute of Human Genetics, University Hospital Heidelberg, 69120 Heidelberg, Germany

**Keywords:** genetic renal disease, model organisms, functional validation, pathophysiology, review

## Abstract

Functional validation is key for establishing new disease genes in human genetics. Over the years, model organisms have been utilized in a very effective manner to prove causality of genes or genetic variants for a wide variety of diseases. Also in hereditary renal disease, model organisms are very helpful for functional validation of candidate genes and variants identified by next-generation sequencing strategies and for obtaining insights into the pathophysiology. Due to high genetic conservation as well as high anatomical and physiological similarities with the human kidney, almost all genetic kidney diseases can be studied in the mouse. However, mouse work is time consuming and expensive, so there is a need for alternative models. In this review, we will provide an overview of model organisms used in renal research, focusing on mouse, zebrafish, frog, and fruit flies.

## Introduction

Identification of new disease genes has been very successful in the last two decades. The main reason is the advent of next-generation sequencing (NGS) technologies that allow the fast and inexpensive sequencing of large numbers of patients. Currently, more than 4000 genes are listed as disease genes in the Online Mendelian Inheritance in Man (OMIM) database, and the list continues to grow [1]. In addition, there is a flurry of genome-wide association studies (GWAS) providing additional associations between gene loci and diseases [2]. Why is it important to discover new disease genes? Besides providing new insights into biological processes, disease gene discovery also improves the quality of life of patients and their families, e. g., by ending “diagnostic odysseys,” reducing unnecessary diagnostic tests, offering prenatal diagnosis options, and sometimes improving medical care for individual patients by a specific therapy. For families with renal disease, in particular, the selection of suitable transplantation donors is also a key outcome of proper genetic diagnostics [3].

One of the biggest challenges in disease gene discovery, however, is to prove causality between a genetic variant and the phenotype of a patient. Exome/whole-genome sequencing approaches typically identify many variants, but deciding which of them is the causative one remains difficult. Bioinformatic filtering tools allow the prioritization of candidate genes. Yet, despite their usefulness, these computational methods alone cannot prove causality for the phenotype of interest. So, unless one is fortunate enough to have discovered a large number of families affected by the same variant or by different variants in the same gene, which nowadays is rare, functional validation will be key for establishing a novel disease gene. And even if genetic causes have been clarified, functional validation may be required to understand the pathophysiology.

Functional validation can be performed in cell culture or model organisms. Through the advent of organoids, cell culture models have received a huge upgrade. However, as organoids were only recently introduced, they have not yet been employed much in human genetics. Hence, this review will give an overview on model organisms used for functional validation in human genetics, with a special focus on genetic renal disease.

**Figure 1:**
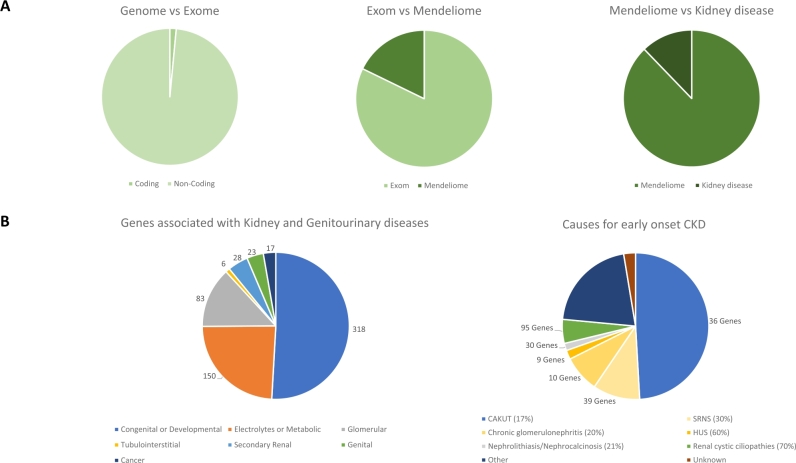
(**A**) Fraction of kidney disease genes in relation to the human genome. About 1.5 % of the entire human genome encodes proteins, referred to as the exome. Of the human exome, 18 % is associated with diseases with an underlying Mendelian inheritance [1]. The term “Mendeliome” includes genes for which a clinical relevance and/or a monogenic inheritance has been proven. From these 4300 genes (Mendeliome), about 600 are currently associated with kidney diseases. (**B**) Different types of kidney diseases and the number of established causal genes. Renal cancer and genitourinary disease are multisystem disorders with secondary renal involvement [5]. In children, the most important genetic causes of CKD are CAKUT, SRNS, and renal ciliopathies [8]. Early-onset CKD is defined as cases with CKD that manifests before the age of 25. A genetic cause has been identified in only 20 % of cases. Currently, 219 genes are known as causative for early-onset CKD. The fraction of causative mutations identified for each diagnostic group and the number of currently known causitive genes are given in parentheses in the figure.

**Figure 2:**
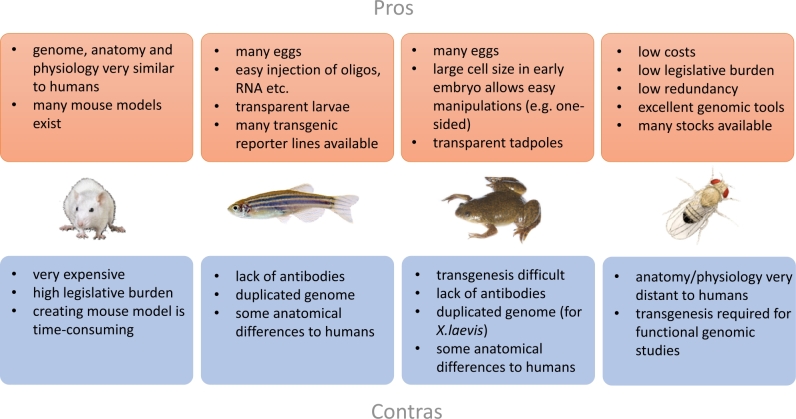
General advantages and disadvantages of the model organisms discussed in this review.

## Overview of different genetic renal diseases

The kidney is a complex organ with more than 25 cell types in humans. It eliminates toxic waste, balances fluid intake and excretion, controls electrolyte homeostasis, and produces essential hormones. Kidney disease is a growing global health burden [4]. However, only little progress has been made in understanding and treating the underlying causes of hereditary or acquired kidney diseases. In most cases of end-stage renal disease (ESRD), the two renal replacement strategies, dialysis and transplantation, are the only treatment options.

More than 600 genes have been associated with monogenic renal disease [5] (see Fig. 1). Most of the major renal disease genes, such as the two autosomal-dominant polycystic kidney disease (ADPKD) genes *PKD1* and *PKD2*, were discovered in the 1980s and 1990s through positional cloning approaches [6], [7]. In the last decade, whole-exome sequencing has been most successful for the discovery of new renal disease genes. Typically, the earlier the disease manifestation, the more likely is a genetic cause. Accordingly, the diagnostic rate of NGS-based genetic testing in patients with chronic kidney disease (CKD) is around 20 % in pediatric [8] and 10 % in adult cohorts [9]. The three main genetic renal diseases in pediatrics will be briefly reviewed below. For a more comprehensive overview of genetic renal diseases, we refer to excellent reviews [8], [10].

### Glomerulopathies

Anomalies affecting the establishment and maintenance of the glomerular filtration barrier composed of the podocyte, the glomerular basement membrane (GBM), and the fenestrated endothelial cells are referred to as glomerulopathies. An important feature is the loss of proteins into the urine (proteinuria). When proteinuria exceeds three grams per day, this is called nephrotic syndrome. Due to their resistance to steroids, the genetic forms are referred to as steroid-resistant nephrotic syndrome (SRNS). SRNS genes include genes encoding components of the slit diaphragm (e. g., *NPHS1*, *NPHS2*), the podocyte cytoskeleton (e. g., *MYO1E*, *ACTN4*, and *INF2*), and membrane proteins involved in the anchoring of podocyte foot processes in the extracellular matrix (e. g., *ITGA3*). Finally, Alport syndrome, which is characterized by defective production of the collagen IV-based GBM, is a major glomerulopathy, often combined with eye and ear abnormalities. Around one-quarter of pediatric proteinuric glomerulopathies are hereditary entities and are also identified in a growing fraction of adult-onset cases [11]. Overall, glomerulopathies account for around 20 % pediatric CKD cases, with SRNS being the cause in most cases (approximately 10 %), followed by chronic glomerulonephritis (8 %), and hemolytic uremic syndrome (2 %) [8], and it is believed that for more than half of them a genetic cause remains to be discovered [11].

### CAKUT

Congenital anomalies of the kidney and urinary tract (CAKUT) are defined as a broad spectrum of renal and urinary tract malformations. Structural anomalies range from vesicoureteral reflux (VUR) and renal hypodysplasia to complete renal agenesis (the most severe). CAKUT occurs in about 1–2 in 500 live births and is responsible more than half of pediatric CKD cases [8]. For isolated and non-isolated CAKUT more than 36 genes have been identified so far, accounting for around 17 % of all cases in patients with early-onset CKD [8]. A similar proportion is explained by copy number variations (CNVs) [12]. Many of the affected genes are involved in renal development-specific transcription (e. g., *PAX2*, *EYA1*, *GATA3*, *SALL1*, *HNF1B*), signaling pathways (e. g., *RET*, *BMP7*, *WNT5A*, *LIFR*), or morphogenetic cellular processes (e. g., *FRAS1*, *ITGA8*, *DACT1*) [13]. The likelihood for a genetic cause is increased with early-onset and severe manifestation, positive family history, and extrarenal symptoms. Yet, the genetic diagnosis of CAKUT has proven to be challenging because of phenotypic heterogeneity and incomplete genetic penetrance. In the majority of CAKUT cases, the etiology seems to be multifactorial, involving polygenic inheritance and environmental and epigenetic factors, which still need to be deciphered to gain deeper understanding of CAKUT disease mechanisms.

### Renal ciliopathies

Primary cilia are present on the apical surfaces of tubular epithelial cells. For decades, their function had remained elusive until it was discovered that many protein products of cystic kidney disease genes localize to primary cilia [14]. ADPKD is the major renal cystic disease in adults, while autosomal-recessive PKD (ARPKD) and nephronophthisis (NPH) are the major pediatric ones. Ciliopathies with renal and extrarenal manifestations include Senior–Loken syndrome, Bardet–Biedl syndrome, and Joubert syndrome.

NPH results in kidney failure by the third decade of life and accounts for 15 % of children with ESRD [15]. Mutations in *NPHP1* are the most common cause of juvenile NPH and represent 20–30 % of cases [16]. Additional 25 known NPHP-related genes account for only a third of diagnosed NPHP families. Model organisms like zebrafish have been widely used to establish these genes as disease genes. Beyond functional validation, studies on these genes have provided important insights into ciliary structure, protein transport in and out of cilia, and signaling pathways such as Hedgehog and Wnt signaling [17].

## Model organisms – general considerations

Model organisms enable experimental interventions that can establish causal mechanisms of gene action. They can also provide unique genetic architectures, such as inbred strains and isogenic lines, that are ideal for investigating genetic interactions with the environment. For an animal to serve as a research model, it must have several characteristics: It must be relatively small and easy to keep. It must also reproduce rapidly. Only if these requirements are met, reliable scientific results can be obtained in the laboratory setting and within a reasonably short time. Also, the requirement for an animal model to provide causality for any candidate gene will depend on the nature of the genetic disorder under investigation. A clinical condition defined on the basis of a biochemical or metabolic deficiency, for example, may simply require assays in a cell culture system that allow for probing the involvement of a variant in the respective pathway activity. By contrast, variants suspected of being causal for developmental, behavioral, or physiological disorders cannot reasonably be validated outside of an *in vivo* context. Here, the nature of the disease may even determine the choice of model organisms and their possibilities to model particular organ pathologies (e. g., epilepsies, skeletal malformations).

Recent advances in genome editing, such as the CRISPR/Cas9 and TALEN systems, have not only facilitated the knock-out of genes but also the introduction of specific genetic variants. This is key because the ideal goal should be to validate the impact of the identified variants and not just to provide support for the role of the gene affected by the variant in a cell or developmental process that might explain the phenotype [18]. This is particularly true when only missense variants in the gene in question were discovered in the patient(s) [19]. Another important consideration in the functional validation approach should also be the mode of inheritance of the studied condition. For example, if the condition is dominant, then the disease phenotype should primarily be assessed in heterozygous animals. Taken together, model organisms can be very suitable to model a candidate gene or variant, but the design of the functional validation experiment is critical and needs to be considered on a case-by-case basis.

For functional validation of candidate genes in kidney diseases, several model organisms have been utilized so far. Mouse and fish have been most commonly used, but alternative models are gaining popularity. So let us take a closer look at these model organisms and their utility for functional validation of renal disease genes, starting with mouse and zebrafish, and then moving to the more uncommon models *Xenopus* and *Drosophila* (see Fig. 2). Other model organisms such as rat, worm, and chicken have also been used in renal research. However, as this has not been done very often in the context of disease gene validation, they will not be covered here.

## Mouse – the allrounder

The mouse (*Mus musculus*) is a very powerful model organism. After a short gestation period of just 3 weeks, three to ten offspring are born. The lifespan is around 2 to 3 years. One great advantage is that almost all human disease genes are present in the mouse. Another advantage is that many tools have already been established. For example, many antibodies against mouse proteins exist, and transcriptomic/proteomic studies are facilitated by the excellent genome annotation. Most importantly, a wide array of innovative genetic technologies has been utilized to produce mouse models to study the function of targeted genes and their involvement in specific diseases.

One of the key advances has been the ability to create transgenic mice, in which a new gene or variant is inserted into the germline DNA. Homologous recombination (awarded with the Nobel Prize in 2007) is even more powerful, because it allows the “knock-out” or “knock-in” of genes in their natural location. Yet, the generation of the desired genotype can be quite time consuming, requiring lengthy breeding strategies. To preserve these extremely valuable strains of mice and to assist in the propagation of strains with poor reproduction, researchers have taken advantage of state-of-the-art reproductive technologies, including cryopreservation of embryos, *in vitro* fertilization, and ovary transplantation. The Jackson Laboratory, a publicly supported national repository for mouse models in Bar Harbor, Maine, has played a crucial role in maintaining and distributing inbred laboratory mice. In fact, the famous “Black 6” or C57BL/6J mouse strain was developed in the early 1920s by the Jackson Laboratory founder C. C. Little [20]. However, despite strong advances in genome editing technology, creating a knock-out or other transgenic animal can still be very laborious. One of the major hurdles is also to obtain approval from authorities to work with mice, let alone the costs associated with mouse cages and maintenance. Also, the exchange of mice between laboratories or from the Jackson Laboratory is tedious, often requiring expensive and lengthy embryo transfer and rederivation.

For renal research, the mouse has been instrumental, resulting in an enormous number of publications and discoveries. The main reason is that the complexity of a human kidney can only properly be mimicked in mammals, allowing the study of a wide variety of genetic kidney diseases, ranging from developmental to glomerular and tubular disorders. In CAKUT modeling, for example, the spatial and temporal expression of candidate genes during renal development is critical and can be addressed with *in situ* hybridization or immunohistochemistry in the developing murine renal system, which is very similar to the human one. In addition, there are a wide variety of Cre recombinase lines available allowing for conditional knock-out of genes in specific renal cell types during development but also in adulthood. Especially, such conditional knock-outs have been used widely for testing the role in podocytes and the ability to produce proteinuria [21].

## Zebrafish – the shooting star

The zebrafish (*Danio rerio*) has emerged as a very important organism to study human biology. Zebrafish matings can produce between 50 and 200 offspring that can grow to adulthood within 3 months. Being a vertebrate, zebrafish have almost all of the same organs and systems as humans, and these can be rapidly analyzed for a phenotype of interest due to their transparent nature in the larval stage. Especially live cell imaging of fluorescent reporters has been used with great success to monitor biological processes at cellular, and even subcellular, resolution in the intact animal. The sequencing of the zebrafish genome has revealed the conservation of around 70 % of all human genes and 82 % of human disease-related genes [22]. Advanced public resources include a centralized database that actively collects and curates the literature (The Zebrafish Information Network, www.zfin.org) as well as public stock centers that distribute mutant and transgenic zebrafish strains (The Zebrafish International Resource Center, www.zebrafish.org). From here, many reporter and other lines can easily be obtained. Therefore, zebrafish are used widely to validate candidate human disease genes and shed light on the pathophysiology of disease. Even drug discovery through compound screening is feasible and can potentially be carried out in a high-throughput manner.

Gene function can be studied rather easily by injecting synthetic RNAs into early zebrafish embryos, generating transgenic zebrafish, or by altering gene function with genome editing technologies, such as the CRISPR/Cas9 system. In human genetics, a typical functional validation assay procedure is to knock down a gene by injecting morpholino RNAs and then rescue the resulting phenotype with wild-type and mutant mRNA. However, recent papers show that when CRISPR/Cas9-induced mutants were examined alongside morpholino morphants, they exhibited a milder phenotype, suggesting that milder phenotypes observed in mutants are thought to be the result of compensatory mechanisms [23], [24]. Another issue is that teleosts like zebrafish underwent genome duplication, which means that the knock-down and knock-out of a gene might be compensated for by a paralogous gene. All of this should be taken into account when performing experiments with morpholino mRNAs or mutants.

So, what about the kidney? In the adult stage, zebrafish have a mesonephric rather than metanephric kidney as found in mammals. In the more studied larval stage, the zebrafish kidney is a pronephric kidney, which is even more basic. Two bilateral glomeruli are linked with two pronephric ducts that have similar genes and functions as the respective segments in the mammalian nephron [25]. For example, while the proximal segments express megalin and cubilin at the apical brush border membrane and are specialized in protein uptake, the distal segment expresses the chloride–protein exchanger Clc-k for pH and chloride balance. The final urine is then directly excreted via the cloacae without any bladder [25].

Many disease genes and processes have been modeled in the zebrafish pronephros. Especially in the ciliopathy field, the pronephros of zebrafish larvae has been quite popular for gene validation [26]. Each cell in the pronephric duct expresses cilia on their apical surfaces, and cilia-related phenotypes, such as renal cysts, can readily be observed in the transparent animal. It has to be taken into account, though, that unlike in the mammalian nephron all cilia in the zebrafish pronephros are motile 9+2 cilia, which are sometimes organized in bundles [27]. These cilia are mainly involved in fluid movement, which could mean that cyst formation in fish may be the result of impaired fluid movement rather than fluid flow sensing and signaling, which have been implicated in human PKD and NPH [28].

## *Xenopus* – the classic one

The clawed frog *Xenopus* was first introduced as an embryological laboratory animal in the early twentieth century, when UK zoologists succeeded in breeding specimens in aquariums. A central feature of this model is the easy access to eggs and embryos: females lay thousands of eggs that are large, with diameters of around 1 mm, allowing for easy microinjection [29]. Even after several cell divisions, specific cells in the early embryo can be injected, which is helpful for creating mosaic tissues. The most famous and celebrated *Xenopus* experiment was John Gurdon’s demonstration of the pluripotency of the somatic nucleus by cloning the very first vertebrate by nuclear transfer into a denucleated fertilized zygote [30]. For this, he received the Nobel Prize 54 years later in 2012. By using *Xenopus* as a model organism, many fundamental cell biological and developmental concepts were generated, most importantly the molecular regulation of the cell cycle [31], the identification of numerous key vertebrate developmental genes [32], and the fundamentals of several signaling pathways, most prominently Wnt and BMP signaling [33].

Two *Xenopus* species are used in the lab: the more traditional *Xenopus laevis* (South African clawed frog), which has been utilized for decades, and the more recently introduced *Xenopus tropicalis* (Western clawed frog) [34]. *X. laevis*, which prefers colder waters of 18 °C, is the product of a fertile hybridization of two species, i. e., contains like zebrafish a duplicated set of genes and chromosomes, while *X. tropicalis* is a genetically diploid species that is raised at temperatures of 24–26 °C. These features have made *X. tropicalis* more attractive in recent years [35], [36].

As aquatic animals, anatomy and physiology are fairly similar to those of the zebrafish kidney. Also here, kidney development proceeds from the very simple pronephros to the more complex mesonephros and the familiar metanephros of mature mammals [29]. In the aquatic tadpoles, the pronephros is the first and fully functional embryonic kidney. Because it consists of one nephron on each side, it is ideally suited to studying the various steps of kidney development from tubulogenesis to segmentation and active filtration. This can be done by unilateral injection using the contralateral side as an internal control [37], [38], [39]. One difference to the zebrafish is that the *Xenopus* glomerulus (also called glomus) does not have a Bowman’s space. Instead, the blood filtrate goes directly into the coelomic cavity, which is drained by the proximal pronephric duct.

CAKUT is so far the most studied renal disease in *Xenopus*. Genes like *GDF6* [40], *DYRK1A* [41], and *NRIP1* [42] are recent examples for functional validation in frog alongside with presentation of patient data. For a more complete list of CAKUT genes whose expression and function have been studied in *Xenopus*, we refer to [43]. *Xenopus* has also been employed for studying NPH. The first report was our study using *Xenopus* to demonstrate a role of *NPHP2*/*INVS* in Wnt signaling [44]. Later, Lienkamp et al. demonstrated the role of *NPHP2*/*INVS* in Wnt-dependent morphogenetic cell movements during tubule elongation [45], suggesting that cyst formation may be the result of early developmental defects. A more recent example is the pronephros phenotype of *NPHP16*/*ANKS6* that has helped to define a specific ciliary module consisting of ANKS6, INVS, NEK8, and NPHP3 [39]. Performing voltage-clamp experiments in *Xenopus* oocytes is another important application of the *Xenopus* system. In this manner, a genotype–phenotype correlation for *CLCNK1* mutations was established for Bartter syndrome type III [46].

Important limitations of the *Xenopus* system are that as an aquatic animal the frog does not require the resorption of water and that most studies are carried out in the pronephros, which is different from the mammalian metanephros. While both these limitations also apply to zebrafish, the generation of transgenic animals has proven difficult in *Xenopus* and is only rarely carried out, which is not the case for all the other model organisms discussed in this review.

## *Drosophila* – the sophisticated one

In the past century, *Drosophila melanogaster* (fruit fly) has been used as a model organism to understand fundamental principles of genetics, developmental biology, immunity, and neuroscience [47]. It is simple to work with, with a relatively short lifecycle/generation time of 10 days, and its small size allows it to be produced in large numbers. The major advantages lie in the low costs, low legislative burden, excellent genome annotation, and vast resources for genomic manipulation. No less than seven Nobel Prizes have been awarded to researchers utilizing the fly as a model. The first one was awarded to T. H. Morgan, who used the fly to prove the chromosomal theory of inheritance showing that the *white* gene resided on the X chromosome. He and his students then went on to define many of the principles of genetics, including the mutagenic effects of X-rays, for which one of the students, H. Muller, also won the Nobel Prize [48].

From these discoveries came the generation of balancer chromosomes, a set of specialized chromosomes that prevent recombination through a series of DNA inversions. These chromosomes permit maintaining complex stocks with multiple mutations on single chromosomes; and because they give rise to obvious phenotypes, such as curled wings, they allow researchers to “see” the genotype without the need for PCR-based genotyping. Building on these foundations, many additional sophisticated biochemical, molecular, and cellular techniques, such as the UAS–GAL4 system, which allows for temporal and spatial gene expression, and *flp*/*FRT*-mediated somatic recombination, for mosaic analyses, were developed. The most famous examples of the feasibility of *Drosophila* in biological research include the identification and cloning of the bithorax complex by E. Lewis [49] and the genome-wide mutational screen carried out by C. Nüsslein-Volhard and E. Wieschaus leading to the discovery of many genes involved directly in regulating embryonic development [50]. For this, Lewis, Nüsslein-Volhard, and Wieschaus shared the Nobel Prize in 1995.

In the last two decades, *Drosophila* has become an important model to dissect and understand the molecular mechanisms that underlie human diseases. When the first 1000 genes registered in OMIM were surveyed for fly orthologs, around 75 % of human disease genes were found to be conserved in *Drosophila* [51]. A more recent study suggests even higher gene conservation and also that genes that are essential in flies are more likely to be associated with human diseases [52]. One very important point is that the fly genome is much less redundant than vertebrate genomes. As there is often only one paralog for one gene, this means that loss-of-function studies are likely to show a phenotype. By integrating techniques to knock out, knock in, knock down, or overexpress endogenous and exogenous proteins in a spatiotemporally controlled manner, Drosophilists can quickly unravel the biological function of a gene or variant of interest *in vivo*. All of this work is made possible due to rich public resources that support fly research, including a centralized database (FlyBase, www.flybase.org) and public stock centers that distribute >100,000 different strains of flies (e. g., the Bloomington Drosophila Stock Center or the Vienna Drosophila Resource Center).

The fly renal system consists of nephrocytes and Malpighian tubules. Nephrocytes are round cells that exist in two populations: around the esophagus (Garland nephrocytes) and parallel to the heart tube (pericardial nephrocytes). For maximal reabsorption of the hemolymph, the surface area is amplified by membrane invaginations that are sealed by slit diaphragms at their outermost edges. The slit diaphragms are composed of nephrin, NEPH1, and podocin orthologs, while the invagination membranes express cubilin and amnionless for protein reabsorption. This is why nephrocytes can be regarded as a hybrid of podocytes and proximal tubular cells [53]. A major difference to vertebrate kidneys is that although nephrocytes filter hemolymph components through their slit diaphragms, the filtrate is directly reabsorbed by the nephrocytes but is not passed on to a tubular system for further modification and excretion. Instead, hemolymph fluid and electrolytes are secreted with great efficiency into the lumen of the Malpighian tubules, which float in the hemolymph without any physical connection to the nephrocytes. The main cell types performing these transport tasks are the principal cells and stellate cells. The principal cells drive electrogenic cation transport through an apical plasma membrane vacuolar ATPase (V-ATPase), contrary to the basolateral Na^+^/K^+^-ATPase in vertebrates, while the stellate cells provide a shunt pathway for chloride and osmosis-driven water movement [53].

The main genetic renal diseases that have been modeled in the fly are glomerulopathies as well as proximal and distal tubulopathies. For example, nephrocyte studies have been used for functional validation of newly discovered SRNS genes, which include *KANK1*/2 [54], *ARHGDIA* [55], *ADCK4* [56], *TBC1D8B* [57], *GAPVD1*, *ANKFY1* [58], and *SGPL1* [59]. Two studies have surveyed all known SRNS genes with fly orthologs through RNA interference (RNAi)-mediated knock-down in nephrocytes, yielding 55 % and 85 %, respectively, of the gene knock-downs tested with nephrocyte phenotypes, such as albumin and silver nitrate uptake [60], [61]. This demonstrates the usefulness of nephrocytes as a model for podocytes, also considering that current cell culture models for podocytes do not allow the study of intact slit diaphragms. A typical functional validation experiment for podocyte diseases would be to express RNAi against the fly ortholog of the candidate gene together with the human cDNA in nephrocytes using the UAS–GAL4 system [19]. Even more elegant is gene replacement in the endogenous locus, as previously done for neurological disorders [62]. However, the generation of transgenic flies with the human reference cDNA to rescue the fly mutations takes up to 6 months and is thus comparably long compared to the RNA injections in frog and fish [47].

## Outlook

While it is getting more and more difficult to identify novel disease genes that affect multiple families, the functional validation possibilities in model organisms are steadily improving. Model organisms could even help to establish disease genes based on singleton cases, which is reflected by the use of fish, worms, and flies by the NIH-funded Undiagnosed Disease Network (UDN) for solving undiagnosed disease in US patients [63]. However, it is important that the experimental work is carried out in a very specific and sensitive manner. Only when the assays are analytically sound and reflect the disease process, then disease gene curation efforts such as those provided by the ClinGen consortium can consider functional studies in model organisms as evidence in favor of or against a gene [64].

So far, there is little experience available for the functional validation of non-coding variants. For renal disease, this has, for example, not yet been done. However, the need for this will grow as whole-genome sequencing is gaining importance. Often such variants affect the expression level of a certain gene, because they lie in enhancer or promoter regions. Comparative genomics approaches that assess conservation between species are important for prioritizing variants. For their functional validation, cells derived from patients will definitely be instrumental, especially when differentiated into a disease-relevant tissue via reprogramming and organoid techniques. But in case of conservation, model organisms will also be helpful for modeling the impact of the non-coding variant *in vivo*. This will most likely also be true for kidney diseases as highlighted in this review by describing the renal systems in four different species. Therefore, it can be expected that model organisms will despite their pros and cons (Fig. 2) stick around for some time to aid us in expanding the list of renal and other disease genes.

## References

[1] Hamosh A (2002). Online Mendelian Inheritance in Man (OMIM), a knowledgebase of human genes and genetic disorders. Nucleic Acids Res.

[2] Tam V (2019). Benefits and limitations of genome-wide association studies. Nat Rev Genet.

[3] Knoers N (2022). Genetic testing in the diagnosis of chronic kidney disease: recommendations for clinical practice. Nephrol Dial Transplant.

[4] Glassock RJ, Warnock DG, Delanaye P (2017). The global burden of chronic kidney disease: estimates, variability and pitfalls. Nat Rev Nephrol.

[5] Rasouly HM (2019). The burden of candidate pathogenic variants for kidney and genitourinary disorders emerging from exome sequencing. Ann Intern Med.

[6] Peters DJ (1993). Chromosome 4 localization of a second gene for autosomal dominant polycystic kidney disease. Nat Genet.

[7] Reeders ST (1985). A highly polymorphic DNA marker linked to adult polycystic kidney disease on chromosome 16. Nature.

[8] Vivante A, Hildebrandt F (2016). Exploring the genetic basis of early-onset chronic kidney disease. Nat Rev Nephrol.

[9] Groopman EE (2019). Diagnostic utility of exome sequencing for kidney disease. N Engl J Med.

[10] Devuyst O (2014). Rare inherited kidney diseases: challenges, opportunities, and perspectives. Lancet.

[11] Sadowski CE (2015). A single-gene cause in 29.5 % of cases of steroid-resistant nephrotic syndrome. J Am Soc Nephrol.

[12] Verbitsky M (2019). The copy number variation landscape of congenital anomalies of the kidney and urinary tract. Nat Genet.

[13] Nigam A, Knoers N, Renkema KY (2019). Impact of next generation sequencing on our understanding of CAKUT. Semin Cell Dev Biol.

[14] Yoder BK, Hou X, Guay-Woodford LM (2002). The polycystic kidney disease proteins, polycystin-1, polycystin-2, polaris, and cystin, are co-localized in renal cilia. J Am Soc Nephrol.

[15] Stokman MF, Saunier S, Benmerah A (2021). Renal ciliopathies: sorting out therapeutic approaches for nephronophthisis. Front Cell Dev Biol.

[16] Braun DA, Hildebrandt F (2017). Ciliopathies. Cold Spring Harb Perspect Biol.

[17] Luo F, Tao YH (2018). Nephronophthisis: a review of genotype-phenotype correlation. Nephrology.

[18] Cox TC (2015). Utility and limitations of animal models for the functional validation of human sequence variants. Mol Genet Genom Med.

[19] Goncalves S (2018). A homozygous KAT2B variant modulates the clinical phenotype of ADD3 deficiency in humans and flies. PLoS Genet.

[20] Little CC (1917). The relation of yellow coat color and black-eyed white spotting of mice in inheritance. Genetics.

[21] Wu F (2007). Conditional targeting in the kidney. Nephron Physiol.

[22] Howe K (2013). The zebrafish reference genome sequence and its relationship to the human genome. Nature.

[23] Rossi A (2015). Genetic compensation induced by deleterious mutations but not gene knockdowns. Nature.

[24] Cardenas-Rodriguez M (2021). Genetic compensation for cilia defects in cep290 mutants by upregulation of cilia-associated small GTPases. J Cell Sci.

[25] Wingert RA, Davidson AJ (2008). The zebrafish pronephros: a model to study nephron segmentation. Kidney Int.

[26] Ross AJ (2005). Disruption of Bardet-Biedl syndrome ciliary proteins perturbs planar cell polarity in vertebrates. Nat Genet.

[27] Zhu P, Qiu Q, Harris PC, Xu X, Lin X (2021). *mtor* haploinsufficiency ameliorates renal cysts and cilia abnormality in adult zebrafish *tmem67* mutants. J Am Soc Nephrol.

[28] Kramer-Zucker AG (2005). Cilia-driven fluid flow in the zebrafish pronephros, brain and Kupffer’s vesicle is required for normal organogenesis. Development.

[29] Blum M, Ott T (2018). Xenopus: an undervalued model organism to study and model human genetic disease. Cells Tissues Organs.

[30] Gurdon JB, Elsdale TR, Fischberg M (1958). Sexually mature individuals of Xenopus laevis from the transplantation of single somatic nuclei. Nature.

[31] Philpott A, Yew PR (2008). The Xenopus cell cycle: an overview. Mol Biotechnol.

[32] De Robertis EM, Larrain J, Oelgeschlager M, Wessely O (2000). The establishment of Spemann’s organizer and patterning of the vertebrate embryo. Nat Rev Genet.

[33] Cruciat CM, Niehrs C (2013). Secreted and transmembrane wnt inhibitors and activators. Cold Spring Harb Perspect Biol.

[34] Tandon P, Conlon F, Furlow JD, Horb ME (2017). Expanding the genetic toolkit in Xenopus: approaches and opportunities for human disease modeling. Dev Biol.

[35] Hellsten U (2010). The genome of the Western clawed frog Xenopus tropicalis. Science.

[36] Session AM (2016). Genome evolution in the allotetraploid frog Xenopus laevis. Nature.

[37] Lienkamp SS (2016). Using Xenopus to study genetic kidney diseases. Semin Cell Dev Biol.

[38] Lienkamp SS (2012). Vertebrate kidney tubules elongate using a planar cell polarity-dependent, rosette-based mechanism of convergent extension. Nat Genet.

[39] Hoff S (2013). ANKS6 is a central component of a nephronophthisis module linking NEK8 to INVS and NPHP3. Nat Genet.

[40] Martens H (2020). Rare heterozygous GDF6 variants in patients with renal anomalies. Eur J Hum Genet.

[41] Blackburn ATM (2020). Correction: DYRK1A-related intellectual disability: a syndrome associated with congenital anomalies of the kidney and urinary tract. Genet Med.

[42] Vivante A (2017). A dominant mutation in nuclear receptor interacting protein 1 causes urinary tract malformations via dysregulation of retinoic acid signaling. J Am Soc Nephrol.

[43] Blackburn ATM, Miller RK (2019). Modeling congenital kidney diseases in Xenopus laevis. Dis Models Mech.

[44] Simons M (2005). Inversin, the gene product mutated in nephronophthisis type II, functions as a molecular switch between Wnt signaling pathways. Nat Genet.

[45] Lienkamp S (2010). Inversin relays Frizzled-8 signals to promote proximal pronephros development. Proc Natl Acad Sci USA.

[46] Seys E (2017). Clinical and genetic spectrum of Bartter syndrome type 3. J Am Soc Nephrol.

[47] Bellen HJ, Yamamoto S (2015). Morgan’s legacy: fruit flies and the functional annotation of conserved genes. Cell.

[48] Muller HJ (1928). The production of mutations by X-rays. Proc Natl Acad Sci USA.

[49] Lewis EB (1982). Control of body segment differentiation in Drosophila by the bithorax gene complex. Prog Clin Biol Res.

[50] Nusslein-Volhard C, Wieschaus E (1980). Mutations affecting segment number and polarity in Drosophila. Nature.

[51] Reiter LT, Potocki L, Chien S, Gribskov M, Bier E (2001). A systematic analysis of human disease-associated gene sequences in Drosophila melanogaster. Genome Res.

[52] Yamamoto S (2014). A drosophila genetic resource of mutants to study mechanisms underlying human genetic diseases. Cell.

[53] Dow JAT, Simons M, Romero MF (2022). Drosophila melanogaster: a simple genetic model of kidney structure, function and disease. Nat Rev Nephrol.

[54] Gee HY (2015). KANK deficiency leads to podocyte dysfunction and nephrotic syndrome. J Clin Invest.

[55] Gee HY (2013). ARHGDIA mutations cause nephrotic syndrome via defective RHO GTPase signaling. J Clin Invest.

[56] Ashraf S (2013). *ADCK4* mutations promote steroid-resistant nephrotic syndrome through CoQ_10_. J Clin Invest.

[57] Kampf LL (2019). *TBC1D8B* mutations implicate RAB11-dependent vesicular trafficking in the pathogenesis of nephrotic syndrome. J Am Soc Nephrol.

[58] Braun DA (2018). Mutations in multiple components of the nuclear pore complex cause nephrotic syndrome. J Clin Invest.

[59] Lovric S (2017). Mutations in sphingosine-1-phosphate lyase cause nephrosis with ichthyosis and adrenal insufficiency. J Clin Invest.

[60] Hermle T, Braun DA, Helmstadter M, Huber TB, Hildebrandt F (2017). Modeling monogenic human nephrotic syndrome in the *Drosophila* garland cell nephrocyte. J Am Soc Nephrol.

[61] Fu Y (2017). A Drosophila model system to assess the function of human monogenic podocyte mutations that cause nephrotic syndrome. Hum Mol Genet.

[62] Lu S (2022). *De novo* variants in *FRMD5* are associated with developmental delay, intellectual disability, ataxia, and abnormalities of eye movement. Am J Hum Genet.

[63] Baldridge D (2021). Model organisms contribute to diagnosis and discovery in the undiagnosed diseases network: current state and a future vision. Orphanet J Rare Dis.

[64] Kanavy DM (2019). Comparative analysis of functional assay evidence use by ClinGen Variant Curation Expert Panels. Gen Med.

